# Acute Neuroinflammatory Response in the Substantia Nigra Pars Compacta of Rats after a Local Injection of Lipopolysaccharide

**DOI:** 10.1155/2018/1838921

**Published:** 2018-05-08

**Authors:** Yazmin M. Flores-Martinez, Manuel A. Fernandez-Parrilla, Jose Ayala-Davila, David Reyes-Corona, Victor M. Blanco-Alvarez, Luis O. Soto-Rojas, Claudia Luna-Herrera, Juan A. Gonzalez-Barrios, Bertha A. Leon-Chavez, Maria E. Gutierrez-Castillo, Irma A. Martínez-Dávila, Daniel Martinez-Fong

**Affiliations:** ^1^Departamento de Fisiología, Biofísica y Neurociencias, Av. Instituto Politécnico Nacional No. 2508, San Pedro Zacatenco, Centro de Investigación y de Estudios Avanzados, 07360 Ciudad de México, Mexico; ^2^Facultad de Ciencias Químicas, 14 Sur y Avenida San Claudio, Benemérita Universidad Autónoma de Puebla, 72570 Puebla, PUE, Mexico; ^3^Departamento de Fisiología, Escuela Nacional de Ciencias Biológicas, Prolongación de Carpio y Plan de Ayala s/n, Santo Tomás, Instituto Politécnico Nacional, 11340 Ciudad de México, Mexico; ^4^Laboratorio de Medicina Genómica, Hospital Regional 1° de Octubre, Avenida Instituto Politécnico Nacional No. 1669, Lindavista, ISSSTE, 07300 Ciudad de México, Mexico; ^5^Departamento de Biociencias e Ingeniería, Centro Interdisciplinario de Investigaciones y Estudios sobre Medio Ambiente y Desarrollo, 30 de Junio de 1520 s/n, La Laguna Ticoman, Instituto Politécnico Nacional, 07340 Ciudad de México, Mexico; ^6^Programa de Doctorado en Nanociencias y Nanotecnología, Av. Instituto Politécnico Nacional No. 2508, San Pedro Zacatenco, Centro de Investigación y de Estudios Avanzados, 07360 Ciudad de México, Mexico

## Abstract

Models of Parkinson's disease with neurotoxins have shown that microglial activation does not evoke a typical inflammatory response in the substantia nigra, questioning whether neuroinflammation leads to neurodegeneration. To address this issue, the archetypal inflammatory stimulus, lipopolysaccharide (LPS), was injected into the rat substantia nigra. LPS induced fever, sickness behavior, and microglial activation (OX42 immunoreactivity), followed by astrocyte activation and leukocyte infiltration (GFAP and CD45 immunoreactivities). During the acute phase of neuroinflammation, pro- and anti-inflammatory cytokines (TNF-*α*, IL-1*β*, IL-6, IL-4, and IL-10) responded differentially at mRNA and protein level. Increased NO production and lipid peroxidation occurred at 168 h after LPS injection. At this time, evidence of neurodegeneration could be seen, entailing decreased tyrosine hydroxylase (TH) immunoreactivity, irregular body contour, and prolongation discontinuity of TH^+^ cells, as well as apparent phagocytosis of TH^+^ cells by OX42^+^ cells. Altogether, these results show that LPS evokes a typical inflammatory response in the substantia nigra that is followed by dopaminergic neurodegeneration.

## 1. Introduction

Neuroinflammation plays a critical role in Parkinson's disease and other neurodegenerative diseases [1, 2]. The main hallmark of neuroinflammation in Parkinson's disease is the presence of activated microglia in the substantia nigra of humans [3] and animal models of that disease [4–6]. Similar to macrophages, activated microglia can phagocytose, present antigens through the major histocompatibility complex (MHC) class II [2, 7], synthesize, and release humoral factors such as cytokines, chemokines, reactive oxygen-nitrogen species, complement cascade proteins, and prostaglandins [8–11]. The tumor necrosis factor- (TNF-) *α*, interleukin- (IL-) 1, and IL-6 transform astrocytes into proliferative immunological cells, recruited in the inflamed brain area [12–15]. The participation of glial cells in the neuroinflammation of Parkinson's disease has been characterized to a large extent in animal models generated by neurotoxins such as 6-hydroxydopamine (6-OHDA), 1-methyl-4-phenyl-1,2,3,6-tetrahydropyridine (MPTP), or rotenone [5, 16–19]. These potent neurotoxins primarily cause the death of dopaminergic neurons, so they have not favored the clarification whether neuroinflammation is the cause or consequence of dopaminergic neurodegeneration. Lipopolysaccharide (LPS) appears to be a neuroinflammatory stimulus more suitable to mimic the acute response of microglia that might also occur in the early stage of Parkinson's disease [20].

LPS is a major component of the outer membrane of gram-negative bacteria and a potent inducer of inflammation via activation of toll-like receptor 4 (TLR4) [21], not only in peripheral tissues and organs [22, 23] but also in the central nervous system (CNS) [24, 25]. Studies using systemic injection [26] or ventricular infusion of LPS [24] in rodents have shown accumulation of activated microglia in various brain nuclei mainly in the substantia nigra, thus suggesting that LPS can be useful to study neurodegeneration as a model of Parkinson's disease [24, 25]. LPS injected directly into the substantia nigra can elicit a strong macrophage/microglial local reaction that is followed by the specific death of nigral dopaminergic neurons, thus suggesting that LPS can cause neuronal cell death indirectly through the inflammatory reaction [25, 27]. A recent study has confirmed the microglial activation in the substantia nigra after the local injection of LPS at a dose of 5 *μ*g/5 *μ*L and demonstrated the mRNA expression of proinflammatory cytokines (TNF-*α* and IL-1*β*) after 7 days of injection, alterations in oxidative stress markers after 14 days postinjection, and apoptosis activation after 21 days of LPS injection [28]. However, those inflammatory variables were evaluated in the whole midbrain and not restrained to the substantia nigra [28]. In addition, the time course of neuroinflammation was studied after the end of acute neuroinflammation, in the same period where the specific neurotoxins also cause neuroinflammation [28]; then, the possibility that neuroinflammation would precede dopaminergic neurodegeneration has not been clarified. Nevertheless, microglial response evaluated through OX42 immunohistochemistry has been shown as early as 6 h after an intrastriatal injection of 22.5 *μ*g of LPS, preceding the dopaminergic neurodegeneration [29]. However, this study did not evaluate any proinflammatory cytokines or astrocyte cell markers [29]. In addition, other studies have shown neither microglial activation in the substantia nigra nor nigrostriatal neurodegeneration, but only transient motor dysfunction, after an intrastriatal administration of 10 *μ*g of LPS [30]. This background information shows that the role of microglia and astrocytes in LPS-induced neuroinflammation is not entirely understood in the substantia nigra [25].

The astrocyte is also another key player in human diseases and animal models of neuroinflammation [31]. Activated astrocytes (reactive astrogliosis) have been shown in different models of chronic demyelinating pathology [32] and of neurotoxin-induced Parkinson's disease in the rat [5, 19]. Cultured astrocytes from the cerebellum of rats with a natural demyelinating disease can produce high levels of nitric oxide (NO) and inducible NO synthase (iNOS) mRNA and protein and release TNF-*α* when stimulated with LPS; those responses are resistant to the inhibitory effect of TGF-*β*1 [33]. Genomic analysis in mice has also suggested that the reactive astrocytes induced by a systemic LPS (5 mg/kg of body weight) administration exhibit a phenotype that may be detrimental [26]. These results suggest that activated astrocytes produce hazardous molecules that can prolong and aggravate neuroinflammation, which eventually will lead to neuronal death. Whether activated astrocytes have a role in the model of intranigral administration of LPS remains unknown.

Dopaminergic neurons of the substantia nigra are particularly vulnerable to neuroinflammation due to internal and external factors that lead to a maintained, elevated mitochondrial oxidant stress [34]. An internal factor, for instance, is the decrease in glutathione levels and glutamylcysteine ligase activity that are the natural antioxidant defenses in neuronal cells [35]. This feature can account for the inefficient neutralization of the nonenzymatic oxidation products of dopamine and the powerful oxidants resulting from Fenton reaction in the presence of iron [36]. An external factor for the vulnerability of dopaminergic neurons is the relatively enriched microglial population in the substantia nigra as compared to other brain regions, which can mount a fast response to the minimum imbalance of oxidative stress [37, 38]. Therefore, the evaluation of the acute stage of neuroinflammation in the substantia nigra should provide insight into the physiopathology of dopaminergic neurodegeneration.

Here, we propose that a single dose of LPS in the substantia nigra will activate local microglia followed by astrocyte activation as a primary event of neuroinflammation and then followed by the dopaminergic neurodegeneration. To test this hypothesis, we injected a single dose of LPS (5 *μ*g/2 *μ*L of endotoxin-free physiological saline solution) into the substantia nigra. Then, we evaluated NO production; lipid peroxidation index; immunoreactivity of microglia (OX42), astrocyte (GFAP), and leucocyte (CD45) markers; and pro- and anti-inflammatory cytokines (TNF-*α*, IL-1*β*, IL-6, IL-4, and IL-10) during the acute phase of neuroinflammation (0 to 96 h). We also evaluated the immunoreactivity to tyrosine hydroxylase (TH), a dopaminergic neuron marker in the substantia nigra. We evaluated the molecular and cellular markers at 168 h after LPS injection to determine the end of acute neuroinflammation. Although using an LPS animal model in the research of Parkinson's disease has been well documented [25], the results presented here emphasize the timing course within the substantia nigra, which add new evidence to support that inflammation is the cause of dopaminergic neurodegeneration. This acute neuroinflammation model will be useful in a fast screening of new anti-inflammatory drugs with potential for Parkinson's disease treatment.

## 2. Materials and Methods

### 2.1. Ethics Statement

The experimental protocol (Permit number 162-15) was approved by the Internal Committee for the Care and Use of Laboratory Animals of the Center for Research and Advanced Studies of the National Polytechnic Institute (Cinvestav-IPN) in accordance with the current Mexican legislation, NOM-062-ZOO-1999 and NOM-087-ECOL-1995 (Secretaría de Agricultura, Ganadería, Desarrollo Rural, Pesca y Alimentación (SAGARPA)). All efforts were made to minimize suffering, and the number of animals used was kept to a minimum by the experimental design.

### 2.2. Animals

Adult male Wistar rats weighing between 210 g and 230 g were used. Five rats per cage (acrylic; 34 cm × 44 cm × 20 cm) were housed at constant room temperature (22°C) and 12 h-12 h light-dark cycle with food and water ad libitum.

### 2.3. Stereotaxic Injection of LPS

The rats were anesthetized with a single dose of ketamine (70 mg/kg) and xylazine (6 mg/kg; intraperitoneally) and fixed in a stereotaxic apparatus (Stoelting, Wood Dale, IL, USA). A single dose of LPS from *Escherichia coli* 055:B5 (5 *μ*g/2 *μ*L of endotoxin-free physiological saline solution; Sigma-Aldrich, St. Louis, MO, USA) [39, 40] was injected into the left substantia nigra. We used the following coordinates: AP, +3.2 mm from the interaural midpoint; ML, +2.0 mm from the intraparietal suture; and DV, −6.5 mm from the dura mater [19]. A micropump Mod. 100 (Stoelting, Wood Dale, IL, USA) maintained the flow rate (0.2 *μ*L/min). After the total dose was injected, the needle was allowed to remain in the brain for 7 min and then was withdrawn in 1 min steps. The mock group was injected with 2 *μ*L of endotoxin-free physiological saline solution into the left substantia nigra. An additional control group was rats with no treatment (Untreated (Ut)).

### 2.4. Body Surface Temperature

A fine thermocouple thermometer (Hanna Instruments, Woonsocket, RI, USA) was attached to an adhesive tape and secured on the ventral surface of the chest to measure body surface temperature. The measurements were made at different times after intranigral injection of LPS (1, 2, 3, 5, 8, 24, 48, 96, and 168 h) in the experimental group and at 8 h in the negative control group (mock rats).

### 2.5. Sickness Behavior

Sickness signs consisting of absent exploration and locomotion, curled body posture, irregular fur, piloerection, and closed eyes were evaluated in the LPS-treated group and control (untreated and mock) groups over time after the intranigral injection of LPS [41, 42]. Measurements were performed while the rats were in transparent cages and scored on a four-point scale: 0 = no signs, 1 = one sign, 2 = two signs, and 3 = three or more signs. The experimenter quantifying the sickness signs were blind to experimental and control conditions. The overall agreement between two “blind” raters was 95%.

### 2.6. Reverse Transcription-Quantitative Polymerase Chain Reaction (RT-qPCR)

Each brain was obtained free of meninges and immediately rinsed with cold PBS. Six 0.5 mm coronal slices of the brain between the anterior border of the pituitary and the anterior border of the cerebellum were obtained using a cold metallic rat brain matrix (Stoelting, Wood Dale, IL, USA). The left substantia nigra was quickly dissected out from every coronal slice in cold conditions using a stereomicroscope (Leica ZOOM 2000, Buffalo, NY, USA) equipped with an especial metallic stage to contain ice. Each left substantia nigra was immediately stored in a respective Eppendorf tube at −70°C until use. Total RNA was isolated from the substantia nigra using Trizol (Invitrogen Corporation, Carlsbad, CA, USA), and then RNA treated with RNase-free DNase I. The reverse transcription was made with SuperScript III reverse transcriptase (200 U) using 3 *μ*g of total RNA and 0.1 mg of oligo dT (Invitrogen Corporation, Carlsbad, CA, USA). The reverse-transcribed product was diluted 4 times with molecular biology-grade water. A 2.5 *μ*L sample of the diluted cDNA was mixed with 2X TaqMan Universal Mastermix and 20X TaqMan gene-specific probe (Applied Biosystems, Foster City, CA, USA) in a final volume of 5 *μ*L. cDNAs were amplified in 45 cycles using a 7900HT Fast Real-Time PCR system (Applied Biosystems, Foster City, CA, USA). The TaqMan gene-specific probes were Rn01525859_g1 for rat TNF-*α*, Rn00580432_m1 for rat IL-1*β*, Rn01410330_m1 for rat IL-6, Rn01456866_m1 for rat IL-4, Rn99999012_m1 for rat IL-10, and Rn00667869_m1 for rat *β*-actin, which were used as internal controls and for normalization. The cycle threshold (Ct) values for *β*-actin and rTNF-*α*, rIL-1*β*, rIL-6, rIL-4, and rIL-10 were measured and calculated by Sequence Detection System software (SDS 2.2; Applied Biosystems, Foster City, CA, USA). The 2^−ΔΔCt^ method was used to calculate the relative transcript levels expressed as fold change for gene expression with respect to each of the probes used [5, 43, 44].

### 2.7. Enzyme-Linked Immunosorbent Assay (ELISA)

The substantia nigra was homogenized with the protein extraction buffer containing 100 mM Tris-HCl (pH 7.4), 750 mM NaCl (sodium chloride), 10 mM EDTA (ethylenediaminetetraacetic acid), 5 mM EGTA (ethylene glycol tetraacetic acid), and protease inhibitors (Roche, Basel, Switzerland) [5, 45]. The samples were centrifuged at 1000*g* for 10 min at 4°C. The supernatant was collected and centrifuged again at 20,000*g* for 40 min at 4°C to remove remaining debris. ELISA was performed using a Milliplex MAP Rat cytokine/chemokine magnetic bead panel kit according to the provider's protocol (RECYTMAG_65K; Millipore, Temecula, CA, USA), and reading was made by using the LUMINEX MAGPIX detection system with xPONET software (Millipore Corporation, Billerica, MA, USA). The sensitivity ranges were 2.4 to 10,000 pg/mL for TNF-*α* and IL-1*β*, 73.2 to 300,000 pg/mL for IL-6, and 7.3 to 30,000 pg/mL to IL-4 and IL-10.

### 2.8. NO Production

The content of nitric oxide (NO) was determined through nitrite (NO_2_^−^) accumulation in the supernatant of homogenized substantia nigra samples using the Griess reagent assay [5, 33, 46]. Briefly, tissue samples were mechanically homogenized in PBS and centrifuged at 20,000*g* for 30 min at 4°C. The colorimetric reaction in 100 *μ*L of the supernatant was initiated by adding 100 *μ*L of the Griess reagent (equal volumes of 0.1% N-(1-naphthyl)ethylenediamine dihydrochloride and 1.32% sulfanilamide in 60% acetic acid). The absorbance of the samples was read at 540 nm with a SmartSpec 3000 spectrophotometer (Bio-Rad, Hercules, CA, USA) and interpolated by using a standard curve of sodium nitrite (NaNO_2_; 1 to 10 *μ*M) to calculate the nitrite content.

### 2.9. Lipid Peroxidation Assay

Lipid peroxidation was measured through malondialdehyde (MDA) and 4-hydroxyalkenal (4-HAE) concentration in the supernatant of homogenized substantia nigra samples using the colorimetric method reported previously [5, 33, 46]. Briefly, the tissue samples were homogenized in PBS and centrifuged at 20,000*g* at 4°C for 40 min. Then, 325 *μ*L of 10.3 mM N-methyl-2-phenylindole diluted in a mixture of acetonitrile : methanol (3 volume : 1 volume) was added to 100 *μ*L of the supernatant. The colorimetric reaction was initiated by the addition of 75 *μ*L of methanesulfonic acid. The reaction mixture was strongly shaken and incubated at 45°C for 1 h and then centrifuged at 1000*g* for 10 min. The absorbance in the supernatant was read at 586 nm with a SmartSpec 3000 spectrophotometer (Bio-Rad, Hercules, CA, USA). The absorbance values were compared to a standard curve from 0.5 to 5 *μ*M of 1,1,3,3-tetramethoxypropane to calculate the content of MDA and 4-HAE in the samples.

### 2.10. Western Blot Analysis

Western blot analysis was performed in substantia nigra homogenates. Total protein was determined using the BCA protein kit (Pierce; Meridian, Rockford, USA). Fifty micrograms of protein per line was run on 12% sodium dodecyl sulfate-polyacrylamide gel electrophoresis and transferred onto PVDF membranes (Bio-Rad Laboratories, Hercules, CA, USA). Blots were blocked with TBS containing 5% skim milk, 1% BSA, and 0.1% Tween 20 and incubated overnight at 4°C with a mouse monoclonal anti-CD11b/c (OX42), a marker for activated microglia (1 : 100; Abcam, Cambridge, UK), and a rabbit polyclonal anti-glial fibrillary acidic protein (GFAP), a marker for astrocytes (1 : 1000; DakoCytomation, Glostrup, Denmark). Membranes were washed and then incubated with the secondary antibodies conjugated with horseradish peroxidase (HRP), either goat anti-mouse IgG (1 : 5000; Zymed, San Francisco, CA, USA) or donkey anti-rabbit IgG (1 : 5000; Zymed, Cambridge, MA, USA) in blocking solution, for 1.5 h with continuous shaking at room temperature. Blots were washed, and the immunolabeled proteins were detected using the ECL Western blotting system and Hyperfilm ECL (Amersham, Buckinghamshire, UK). To normalize the total amount of protein per lane, membranes were stripped and incubated with a mouse monoclonal antibody against *β*-actin (1 : 500; Cinvestav, Mexico) [47], followed by a HRP-conjugated goat anti-mouse (1 : 6000; Zymed, San Francisco, CA, USA) following the same procedure of luminescence detection.

### 2.11. Immunostaining Techniques

The presence of microglia, astrocytes, and dopaminergic neurons was shown by double immunofluorescence techniques using the procedure described previously [5, 48]. The rats were deeply anesthetized with sodium pentobarbital (50 mg/kg intraperitoneally) and perfused through the ascending aorta with 30 mL of PBS, followed by 100 mL of 4% paraformaldehyde in PBS. The brain was then removed and maintained in the fixative for 24 h at 4°C. After an overnight incubation in PBS containing 30% sucrose at 4°C, the brain was frozen and then sectioned. Briefly, serial coronal sections of 30 *μ*m thickness were cut using a sliding microtome with a freezing stage (Leica SM1100, Heidelberg, Germany) and consecutively collected in 6 wells, using only the slices in one well for the analysis. The slices were rinsed with PBS for 5 min, permeabilized with PBS-0.1% Triton for 20 min, and incubated with 1% BSA in PBS-0.1% Triton for 30 min to block unspecific binding sites. The primary antibodies were mouse monoclonal anti-tyrosine hydroxylase (TH) (clone TH-2) (1 : 1000; Sigma-Aldrich, St. Louis, MO, USA), rabbit polyclonal anti-TH (1 : 1000; Millipore, Temecula, CA, USA), mouse monoclonal anti-CD11b/c (OX42) (1 : 200; Abcam, Cambridge, UK), mouse anti-CD45 (BD Bioscience, USA), and rabbit polyclonal anti-GFAP (1 : 500; DakoCytomation, Glostrup, Denmark). The secondary antibodies were Alexa Fluor 488 chicken anti-mouse H+L IgG (1 : 300; Invitrogen Molecular Probes, Eugene, Oregon, USA), Alexa Fluor 488 chicken anti-rabbit H+L IgG (1 : 300; Invitrogen Molecular Probes, Eugene, Oregon), Texas red horse anti-mouse H+L IgG (1 : 900; Vector Laboratories, Burlingame, CA, USA), and Texas red goat anti-rabbit H+L IgG (1 : 900; Vector Laboratories, Burlingame, CA, USA). The slices were washed with PBS and mounted on glass slides using VECTASHIELD (Vector Laboratories, Burlingame, CA, USA). Fluorescence images were obtained with a Leica DMIRE2 microscope, using 20x and 40x objectives and filters K3 for Alexa Fluor 488 (green fluorescence) and TX2 for Texas red (red fluorescence). The images were digitized with a Leica DC300F camera (Nussloch, Germany). A multispectral confocal laser scanning microscope (TCS SPE; Leica, Heidelberg, Germany) was used to analyze through a 100x oil-immersion objective the double immunofluorescence against TH-OX42 and TH-GFAP at excitation-emission wavelengths of 488–522 nm (green channel) and 568–635 nm (red channel). Their consecutive 1 *μ*m optical sections were also obtained in the Z-series (scanning rate 600 Hz). The images were acquired using LAS AF software (Leica Application Suite; Leica Microsystems, Nussloch, Germany).

TH immunohistochemistry was made after depletion of endogenous peroxidase using PBS-0.3% Triton X-100 solution containing 3% hydrogen peroxide and 10% methanol at room temperature. The primary antibody was a mouse monoclonal anti-TH clone TH-2 (1 : 1000; Sigma-Aldrich, St. Louis, MO, USA), and the secondary antibody was a horse biotinylated anti-mouse H+L IgG (1 : 200; Vector Laboratories, Burlingame, CA, USA). The immunohistochemical staining was developed using the avidin-biotin-peroxidase complex (1 : 10; ABC Kit; Vector Laboratories, Burlingame, CA, USA) and 393-diaminobenzidine (DAB; Sigma-Aldrich, St. Louis, MO, USA) [19]. After the immunohistochemistry, the slides were stained with hematoxylin-eosin (H&E) and then were mounted on glass slides using Entellan (Merck KGaA, Darmstadt, Germany). Finally, the slides were then examined with a light microscope equipped with 5x and 63x oil-immersion objectives (Leica Microsystems, Nussloch, Germany).

### 2.12. Statistical Analysis

All results were expressed as the mean ± standard deviation (SD) values at least from 3 independent experiments (*n* = 3). The following statistical tests to analyze the difference among groups were used: repeated-measures two-way ANOVA and Bonferroni post hoc test for temperature, sickness behavior, nitrites, and lipid peroxidation qPCR and analysis of IF area density, repeated-measures one-way ANOVA, and Newman-Keuls post hoc test for OX42 and GFAP Western blot and ELISA results. GraphPad Prism 5.0 software (GraphPad Software Inc., La Jolla, CA, USA) was used for statistical analysis. The accepted significance was at *P* < 0.05.

## 3. Results

### 3.1. Time Course of Fever and Sickness

**Figure 1:**
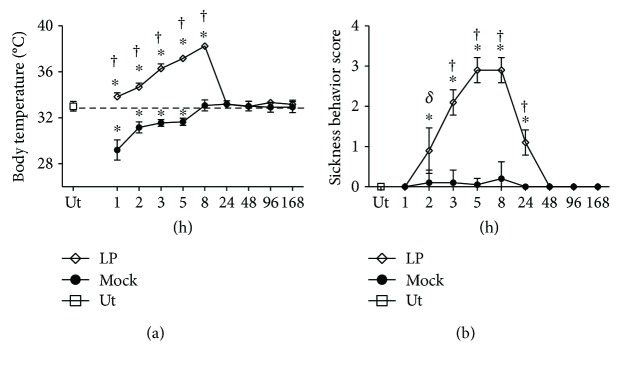
Clinical evolution after a single injection of LPS in the substantia nigra of the rat. (a) Fever. (b) Sickness behavior. Ut = untreated control rats. Mock = rats injected with the vehicle (2 *μ*L of endotoxin-free physiological saline solution) in the left substantia nigra. All values represent the mean ± SD (*n* = 45). ^∗^ *P* < 0.001 when compared with the untreated control group. ^*δ*^ *P* < 0.05 or ^†^ *P* < 0.001 when compared with the respective mock. Repeated-measures two-way ANOVA and Bonferroni post hoc test.

As systemic manifestations of LPS-induced neuroinflammation, the feverish reaction (*n* = 45 rats) and external signs of sickness (*n* = 45 rats) were measured over time (1, 2, 3, 5, 8, 24, 48, 96, and 168 h) after the intranigral injection of LPS. The untreated control rats maintained their body temperature at 32.65 ± 0.75°C, whereas the rats injected with LPS gradually increased their body temperature to a maximum of 38.25 ± 0.15°C detected at 8 h postinjection (Figure 1(a)). After 24 h, the body temperature was maintained at 32.7 ± 0.9°C until 168 h, the end of the experiment (Figure 1(a)). The mock rats (intranigrally injected with 2 *μ*L of endotoxin-free physiological saline solution) showed a body temperature as low as 29.20 ± 0.82°C (1 h) and 31.16 ± 0.45°C (2 h) because of the anesthetic effect [49]. After that, the temperature value attained was 32.65 ± 0.85°C in the untreated control group (Figure 1(a)).

There were no sickness signs in the mock group (*n* = 45 rats) as compared with the untreated control rats, except for slightly irregular fur in 1% of mock rats (score = 1) at 8 h after the vehicle injection (Figure 1(b)). When compared with the untreated controls and mock groups, the rats intranigrally injected with LPS exhibited clear signs of sickness that followed the time course of fever. The maximum score was reached at 8 h postinjection when the rats presented adynamia (absence of locomotion and exploration), curled body posture, closed eyes, and piloerection (Figure 1(b)). The sickness signs varied during their time course, although their score was similar. At 5 h after LPS injection, the predominant signs were the absence of locomotion and exploration, curled body posture, and piloerection, and only closed eyes were observed in 1% of the animals (Figure 1(b)). At 2 and 24 h after LPS injection, the score was 2, but the signs were different in those times. At 2 h, there was no locomotion, exploration, and curled body posture, whereas at 24 h, the locomotion and exploration were recovered, but irregular fur with a slight degree of piloerection was seen in 1% of the rats. At 3 h after LPS injection, the obvious signs were adynamia and curled body posture (Figure 1(b)).

### 3.2. Time Course of Microglial Activation

**Figure 2:**
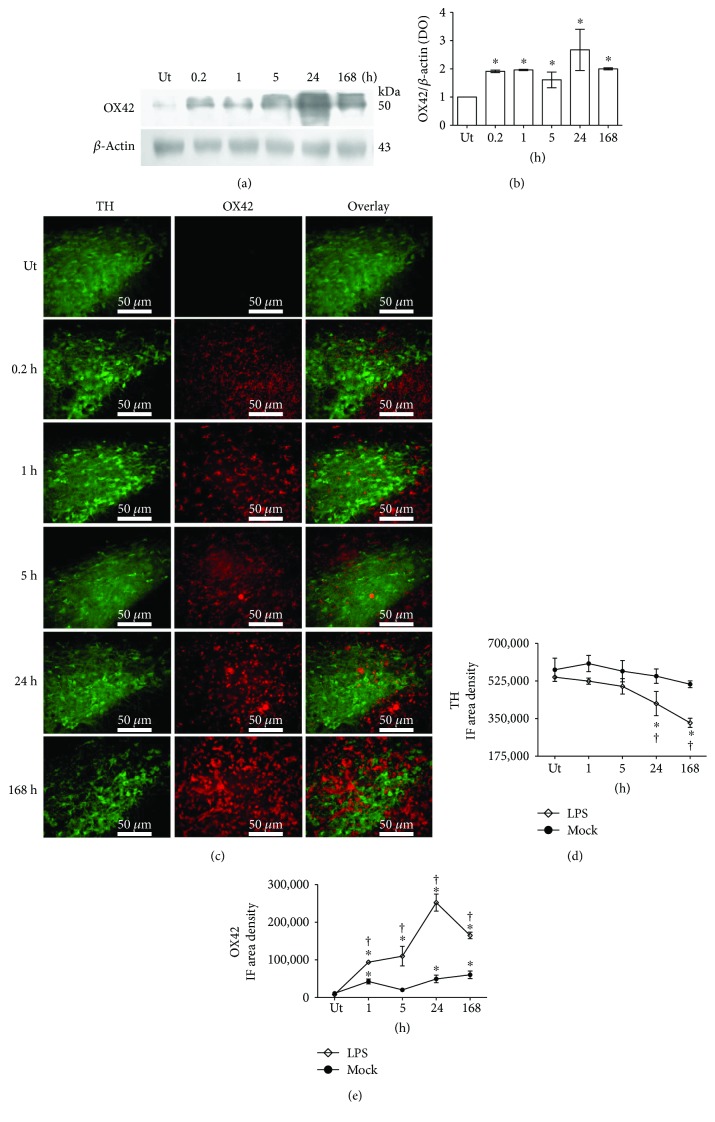
Time course of microglial activation. (a) A representative photograph of a Western blot membrane showing the electrophoretic fractionation of OX42 and *β*-actin from substantia nigra homogenates of LPS-treated rats and untreated (Ut) control rats. The numbers indicate the time of evaluation. (b) Graph of densitometry analysis showing the normalized values of OX42 bands concerning *β*-actin bands. The values represent the mean ± SD (*n* = 3 independent rats in each time of each experimental condition). ^∗^ *P* < 0.001 when compared with the untreated control group using repeated-measures one-way ANOVA and Newman-Keuls post hoc test. (c) Representative micrographs of the double immunofluorescence of TH and OX42 in the substantia nigra of untreated (Ut) control rats and rats at different times after LPS injection that were taken at 3.8 mm from the interaural midpoint on the dorsal-ventral axes of the rat brain atlas by Paxinos and Watson [50]. The numbers at the left side of micrographs indicate the time of evaluation. Immunofluorescence (IF) area density for TH (d) and OX42 (e) was determined using ImageJ software v.1.46r (National Institutes of Health, Bethesda, MD). The TH and OX42 values for the mock rats correspond to the quantification in Supplementary Figure 1. All values represent the mean ± SD (*n* = 3 independent rats in each time of each experimental condition). ^∗^ *P* < 0.001 when compared with the untreated control group of the respective immunostaining. ^†^ *P* < 0.001 when compared with the respective mock group. Repeated-measures two-way ANOVA and Bonferroni post hoc test.

OX42 immunodetection by Western blot (*n* = 3 rats for each time) and double immunofluorescence with TH (*n* = 3 rats for each time) was used to analyze the time course (0.2, 1, 5, 24, and 168 h) of microglial activation in the substantia nigra after LPS injection. The basal levels of OX42 immunoreactivity in the untreated control rats were low and normalized concerning *β*-actin in Western blot assays (Figures 2(a) and 2(b)). Immunofluorescence assays showed scarce OX42 immunoreactivity in the substantia nigra pars compacta of both untreated and mock groups (Figures 2(c) and 2(e) and Supplementary Figure 1). OX42 immunoreactivity increased immediately after the LPS injection, reached a maximum value at 24 h, and still was high at the end of the experiment (168 h) as shown by both immunodetection techniques (Figures 2(a)–2(e)). A significant increase in OX42 immunoreactivity was only detected in the mock condition when compared with the untreated condition at 168 h after the vehicle injection (Figure 2(e) and Supplementary Figure 1), but the increase in the LPS group was twice greater than that in the mock group and was statistically significant (Figure 2(e)). At this time, a significant 39% decrease in the number of TH-immunoreactive cells occurred with respect to the untreated control and mock condition (Figures 2(c) and 2(d) and Supplementary Figure 1), suggesting neurodegeneration of dopaminergic neurons (Figures 2(c) and 2(d)).

**Figure 3:**
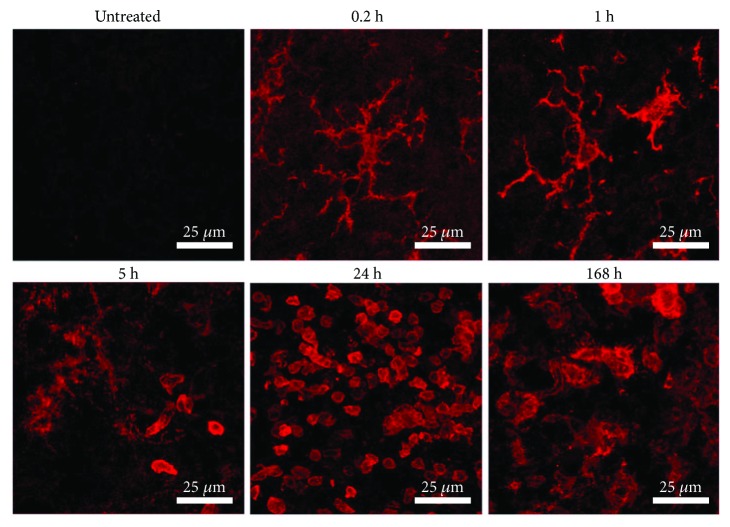
Morphological changes during activation of microglia in the substantia nigra after LPS exposure. Representative confocal micrographs of OX42 immunofluorescence in the substantia nigra of untreated control rats and rats at different times after LPS injection.

It is interesting to notice the morphological changes of microglia as the time elapses after LPS exposure (Figure 3). The absence of OX42-immunoreactive cells in the untreated condition suggests the resting or quiescent condition of microglia. At 12 minutes after LPS injection (0.2 h), the cells exhibited a strong OX42 immunoreactivity and a robust branched morphology, with long thick branches, as well as a regular and slightly enlarged soma (Figure 3). After 1 h, two types of morphology were observed (Figure 3). One type consists of long, thick branching and a well-delimited, wide soma and nucleus (Figure 3). The second type consists of short, stout branches and a larger soma and nucleus. From 5 to 24 h after LPS injection, the reactive-state, round-shape cells with retracted processes and enlarged body, also referred to as the amoeboid form, can be observed (Figure 3). At 168 h after LPS injection, the OX42-immunoreactive cells exhibited a round, irregular, and larger shape than the amoeboid cells suggestive of the phagocytic state (Figure 3).

### 3.3. Time Course of Astrocyte Activation

**Figure 4:**
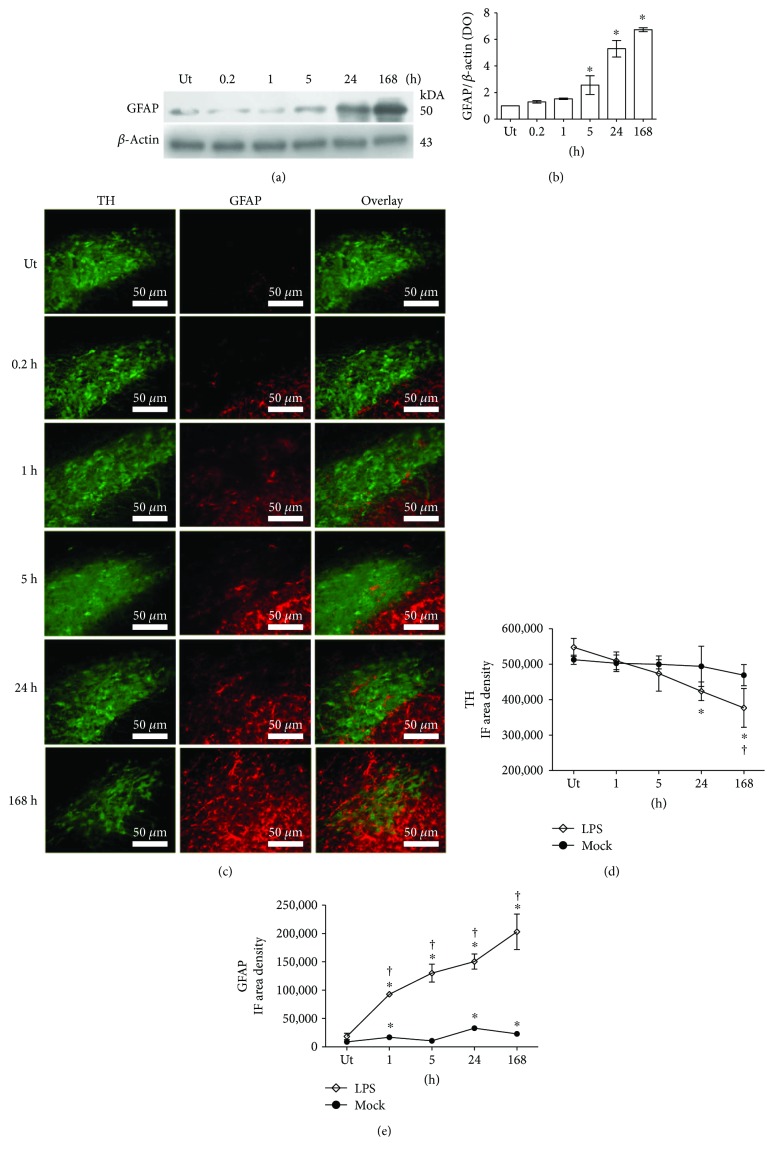
Time course of astrocyte activation. (a) A representative photograph of a Western blot membrane showing the electrophoretic fractionation of GFAP and *β*-actin from substantia nigra homogenates of LPS-treated rats and untreated (Ut) control rats. The numbers indicate the time of evaluation. (b) Graph of densitometry analysis showing the normalized values of GFAP bands with respect to *β*-actin bands. The values represent the mean ± SD (*n* = 3 independent rats for each time of each experimental condition). ^∗^ *P* < 0.001 when compared with the untreated control group using repeated-measures one-way ANOVA and Newman-Keuls post hoc test. (c) Representative micrographs of the double immunofluorescence of GFAP and TH in the substantia nigra of untreated (Ut) control rats and rats at different times after LPS injection that were taken at 3.7 mm from the interaural midpoint on the dorsal-ventral axis of the rat brain atlas by Paxinos and Watson [50]. The numbers at the left side of micrographs indicate the time of evaluation. Immunofluorescence (IF) area density for TH (d) and GFAP (e) was determined using ImageJ software v.1.46r (National Institutes of Health, Bethesda, MD). The TH and GFAP values for the mock rats correspond to the quantification in Supplementary Figure 2. All values represent the mean ± SD (*n* = 3 independent rats in each time and each experimental condition). ^∗^ *P* < 0.001 when compared with the untreated control group of the respective immunostaining. ^†^ *P* < 0.001 when compared with the respective mock group. Repeated-measures two-way ANOVA and Bonferroni post hoc test.

GFAP immunodetection by Western blot (*n* = 3 rats for each time) and double immunofluorescence with TH (*n* = 3 rats for each time) was used to analyze the time course (0.2, 1, 5, 24, and 168 h) of astrocyte activation in the substantia nigra after the LPS injection. Contrary to OX42 immunoreactivity, the Western blot analysis showed that the increase in GFAP immunoreactivity was belated and with statistical significance since 5 h following LPS injection with respect to the basal levels of untreated control rats (Figures 4(a) and 4(b)). GFAP immunoreactivity continued increasing until the end of the study (Figures 4(a) and 4(b)). The immunofluorescence assay agrees with the time course of GFAP immunoreactivity shown by Western blot analysis and revealed details on changes of localization of the GFAP-immunoreactive cells (Figures 4(c) and 4(e)). GFAP-immunoreactive cells were scarce in the substantia nigra of untreated control rats (Figures 4(c) and 4(e)). After LPS administration, GFAP-immunoreactive cells started appearing in the pars reticulata of the substantia nigra (1 h) and then in the pars compacta (24 h). At the end of the study, the GFAP-immunoreactive cells were abundant in both the pars compacta and pars reticulata of the substantia nigra (Figures 4(c) and 4(e)). These results suggest that the astrocytes of the substantia nigra are first activated in the pars reticulata and then recruited in the pars compacta where the dopaminergic neurons dwell. The vehicle injection increased GFAP-immunoreactive cells, which was significant with the untreated controls, but the increase in the LPS group was much greater than that in the mock group and was statistically significant (Figures 4(c) and 4(e) and Supplementary Figure 2). The significant decrease in TH-immunoreactive cells in the LPS group was also confirmed (Figures 4(c) and 4(d)).

**Figure 5:**
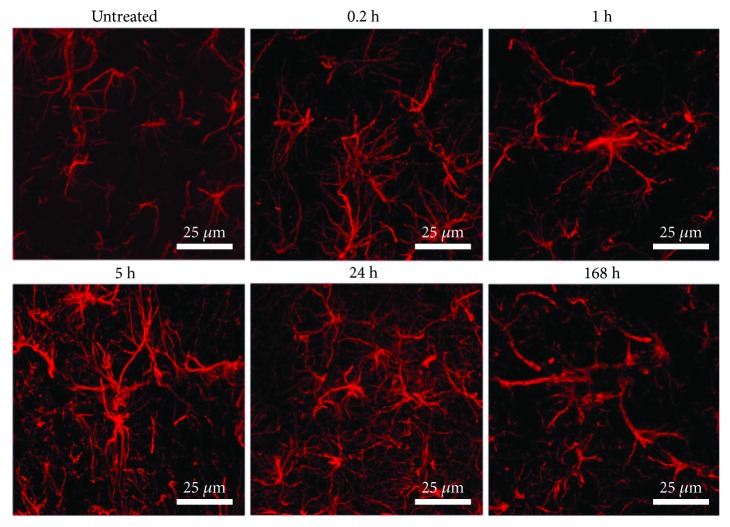
Morphological changes during activation of astrocytes in the substantia nigra after LPS exposure. Representative confocal micrographs of GFAP immunofluorescence in the substantia nigra of untreated control rats and rats at different times after LPS injection.

The morphological changes in astrocytes were less dramatic than those in microglia (Figure 4). Because GFAP-immunoreactive cells were rarely observed in the substantia nigra pars compacta of untreated control rats, their morphological details were shown in GFAP-immunoreactive cells in the substantia nigra pars reticulata (Figure 5). In basal conditions, the GFAP-immunoreactive cells were scarce with small soma and few thin branches (Figure 5). The changes induced by LPS injection mainly consisted of an increase in the cell number, in the GFAP-immunoreactivity intensity, and in the number of branching, which was long and robust (Figure 5). These changes were present until the end of the study.

### 3.4. Nitrosative and Oxidative Stress

**Figure 6:**
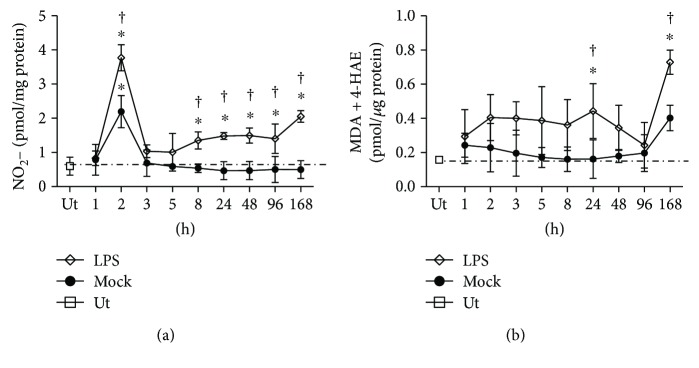
Nitrosative and oxidative stress in the substantia nigra after LPS local injection. (a) Nitrosative stress was evaluated through the levels of nitrites. (b) Oxidative stress (lipid peroxidation) was evaluated through the levels of malondialdehyde (MDA) + 4-hydroxyalkenals (4-HAE). Ut = untreated control rats. Mock = rats injected with the vehicle (2 *μ*L of endotoxin-free physiological saline solution) in the left substantia nigra. The values represent the mean ± SD from 5 rats for each time and each experimental condition. ^∗^ *P* < 0.001 when compared with the untreated control group. ^†^ *P* < 0.001 when compared with the respective mock group. Repeated-measures one-way ANOVA and Bonferroni post hoc test.

We evaluated nitrite concentration as a marker of nitrosative stress (*n* = 5 rats for each time) and MDA + 4-HAE levels as a marker of oxidative stress (*n* = 5 rats for each time). Mock values did not show statistical significance when compared with those of untreated group stress. As compared with the untreated control group, a significant 3.8-fold increase in nitrite levels was observed at 2 h after LPS injection and a second 2.0-fold increase from 8 h to 168 h (Figure 6(a)). The mock rats show a 2.0-fold increase in nitrite levels at 2 h after injection when compared with the untreated control group, but that increase was significantly lesser than that caused by LPS at the same time (Figure 6(a)). Different from nitrosative stress, lipid peroxidation was only significant at 168 h after LPS injection with respect to the untreated control group, suggesting that lipid peroxidation follows the acute neuroinflammation (Figure 6(b)).

### 3.5. Proinflammatory and Anti-Inflammatory Cytokines

**Figure 7:**
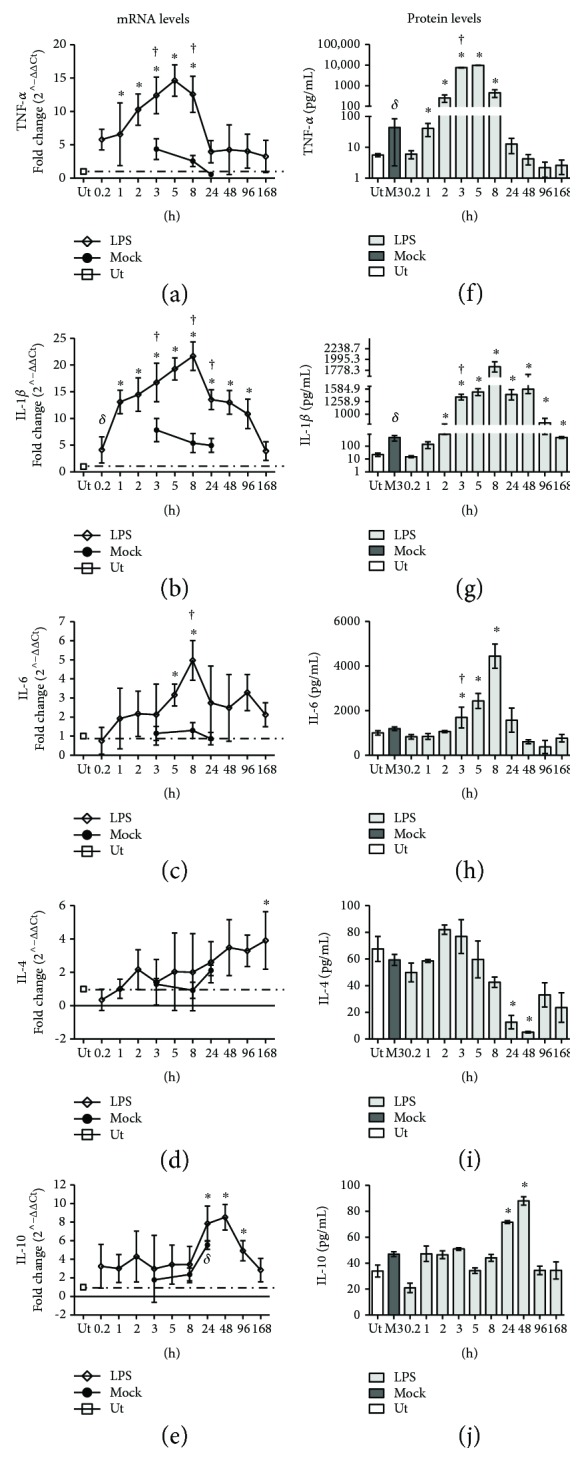
Levels of cytokines in the substantia nigra after LPS local injection. qPCR (a–e) and ELISA (f–j) were used to measure mRNA and protein levels, respectively, for TNF-*α*, IL-1*β*, and IL-6 (proinflammatory cytokines) and IL-4 and IL-10 (anti-inflammatory cytokines). Ut = untreated control rats. Mock = rats injected with the vehicle (2 *μ*L of endotoxin-free physiological saline solution) in the left substantia nigra. All values represent the mean ± SD (*n* = 4 independent rats in each time of each experimental condition). ^*δ*^ *P* < 0.05 or ^∗^ *P* < 0.001 when compared with the untreated control group. ^†^ *P* < 0.001 when compared with the respective mock group. Repeated-measures one-way ANOVA and Newman-Keuls post hoc test for ELISA and repeated-measures two-way ANOVA and Bonferroni post hoc test for the qPCR assay.

**Figure 8:**
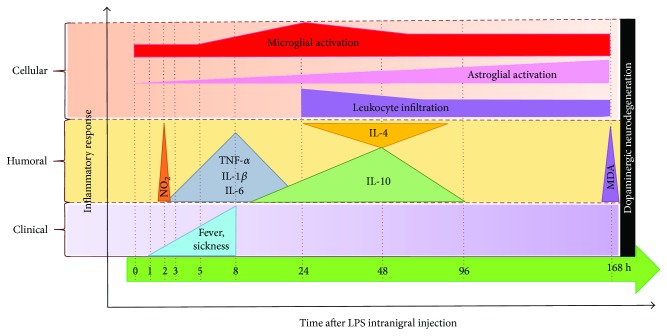
Schematic summary of LPS-induced acute neuroinflammation in the substantia nigra of the rat.

Three proinflammatory cytokines (TNF-*α*, IL-1*β*, and IL-6) and two anti-inflammatory cytokines (IL-4 and IL-10) were evaluated in the substantia nigra through ELISA and qPCR (*n* = 4 rats for each time and each experimental condition; Figure 7). The LPS intranigral injection significantly increased mRNA levels of the three proinflammatory cytokines, but the onset and the peak were different for each proinflammatory cytokine (Figures 7(a)–7(c)). TNF-*α* and IL-1*β* mRNA levels were significant at early times and were maximum at 5 h (Figures 7(a) and 7(b)), followed by IL-6 mRNA levels that were maximum at 8 h (Figure 7(c)), when compared with those of the untreated and mock controls. After that, TNF-*α* and IL-1*β* mRNA levels decreased to reach the basal levels at 24 h; only IL-1*β* mRNA levels remained significantly increased up to 168 h (Figure 7(b)). mRNA levels of the two anti-inflammatory cytokines were significant only at late times when compared with those of the untreated controls: at 168 h, IL-4, and from 24 to 96 h, IL-10 (Figures 7(d) and 7(e)). We found that NO production precedes the increase in proinflammatory cytokine levels and that the clinical effect (fever and sickness behavior) was associated with the time course of proinflammatory cytokines (Figures 1, 6, and 8). Since the vehicle injection neither increased NO production nor elicited clinical manifestations (i.e., fever and sickness behavior) over time, the effect of the vehicle on cytokine mRNA levels was only measured in the period of the maximum increase in mRNA levels in the LPS groups. There was no statistical difference from the untreated controls (Figures 7(a)–7(e)). The basal levels of the three proinflammatory cytokines and the two anti-inflammatory cytokines were, in pg/mL, 5.58 ± 0.80 (TNF-*α*), 22.14 ± 7.72 pg/mL (IL-1*β*), 1001.70 ± 144.01 (IL-6), 67.58 ± 12.80 pg/mL (IL-4), and 33.89 ± 11.21 (IL-10). The LPS intranigral injection significantly increased the basal levels of TNF-*α*, IL-1*β*, IL-6, and IL-10 with a time course similar to that of their respective transcripts (Figures 7(a)–7(d) and 7(f)–7(i)). IL-4 decreased at 24 and 48 h after LPS injection (Figure 7(f)), although its transcript levels were not significantly different over time (Figure 7(e), suggesting posttranscriptional regulation for this anti-inflammatory cytokine. Because the time course of protein levels was similar to that of transcript levels, the vehicle effect on cytokine protein levels was only determined at 3 h postinjection when qPCR showed the maximum increase in the mock group (Figures 7(a)–7(e)). A statistically different increase only occurred in TNF-*α* and IL-1*β* of the mock group with respect to the basal values, but such increase was statically different and 55% lower than that in the respective LPS group (Figures 7(f)–7(h)).

### 3.6. Apparent Microglial Phagocytosis of Damaged Dopaminergic Neurons

**Figure 9:**
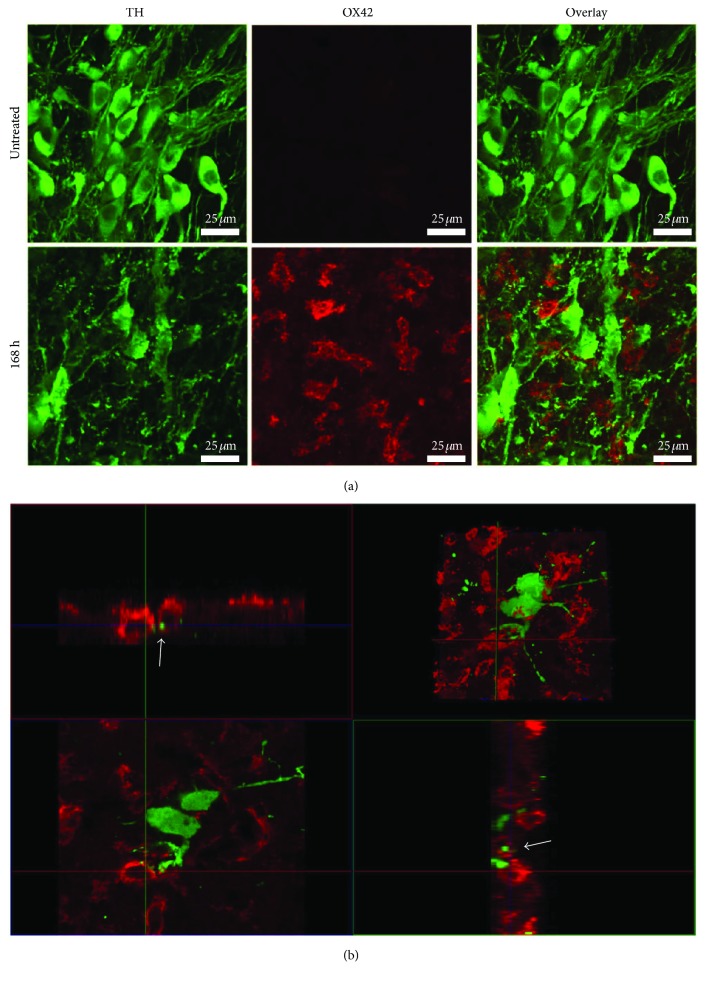
Apparent phagocytosis of damaged dopaminergic neurons by microglia. Representative confocal micrographs of TH and OX42 double immunofluorescence. (a) Integrated projections from x-y optical stacks. (b) Orthogonal projections from a 1 *μ*m z-confocal optical section. The arrows show a green fluorescence dot (TH immunoreactivity) surrounded by a red fluorescence ring (OX42). The right top panel corresponds to the integrated image where the orthogonal analysis was performed.

Confocal analysis with orthogonal projections was used to evaluate whether OX42^+^ cells (microglia) might engulf damaged TH^+^ cells (dopaminergic neurons) in the substantia nigra at 168 h after LPS local administration. The substantia nigra pars compacta of untreated control rats is characterized by the absence of active microglia and normal morphology of dopaminergic neurons with well-defined soma and continuous prolongations (Figure 9(a)). At 168 h after LPS administration, irregular and large OX42^+^ cells are present in tight contact with harmed TH^+^ cells (Figure 9(a)). At this time, evidence of neurodegeneration could be seen, entailing the decreased number of TH^+^ cells (Figures 2(d), 4(d), and 9(a)), irregular body contour, unidentifiable nuclear area, and scarce and discontinuous prolongations. Also, there was evidence of apparent phagocytosis of TH^+^ cells by OX42^+^ cells (Figure 9(a)). This suggestion is further reinforced by the confocal orthogonal views that show TH^+^ cell fragments being encircled by OX42^+^ cell prolongations (Figure 9(b)). These results suggest that acute neuroinflammation by LPS local injection can lead to dopaminergic neurodegeneration in the substantia nigra.

### 3.7. Leukocyte Infiltration

**Figure 10:**
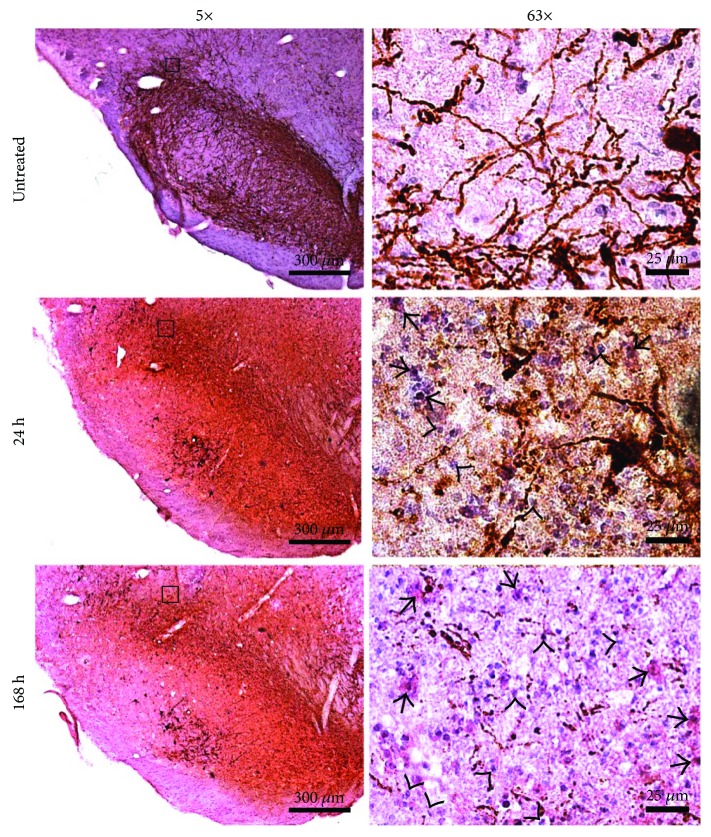
Leukocyte infiltration. Representative micrographs showing the H&E staining and TH immunohistochemistry of the substantia nigra of untreated control rats and experimental rats at 24 and 168 h after LPS injection. The micrographs were taken at 3.2 mm from the interaural midpoint on the dorsal-ventral axis of the rat brain atlas by Paxinos and Watson [50]. The black square on 5x micrographs indicates the area where 63x amplification was taken. Arrowheads indicate leukocyte infiltration, and arrows indicate microglia/macrophages.

The H&E staining in combination with TH immunohistochemistry of the untreated substantia nigra showed the presence of TH^+^ and the absence of infiltrating cells (Figure 10). At 24 and 168 h after LPS injection, two kinds of infiltrating cells can be observed in the cerebral parenchyma: (1) macrophage-like cells with an elongated cytoplasm and a large, eccentric nucleus known as “rod cells” (Figure 10), a characteristic of macrophages and active microglia [51], and (2) leukocyte-like cells characterized by a small, regularly round basophilic cytoplasm and a well-defined large nucleus (Figure 10). The TH immunochemistry staining, besides its usefulness to delimit the substantia nigra compacta, confirmed the findings of the double immunofluorescence, that is, a decrease in TH immunoreactivity and irregular TH^+^ cells suggesting damage of the dopaminergic neuron population (Figure 10). It is interesting to note that a large number of TH^+^ neurons are clearly seen in the substantia nigra pars reticulata at 24 and 48 h after LPS injection, suggesting that those neurons are more resistant to the neuroinflammation elicited by LPS in comparison to those of the substantia nigra compacta (Figures 10(c) and 10(e)). The expression of calretinin in those neurons might explain the resistance to neuroinflammation as occurring for 6-OHDA [52].

**Figure 11:**
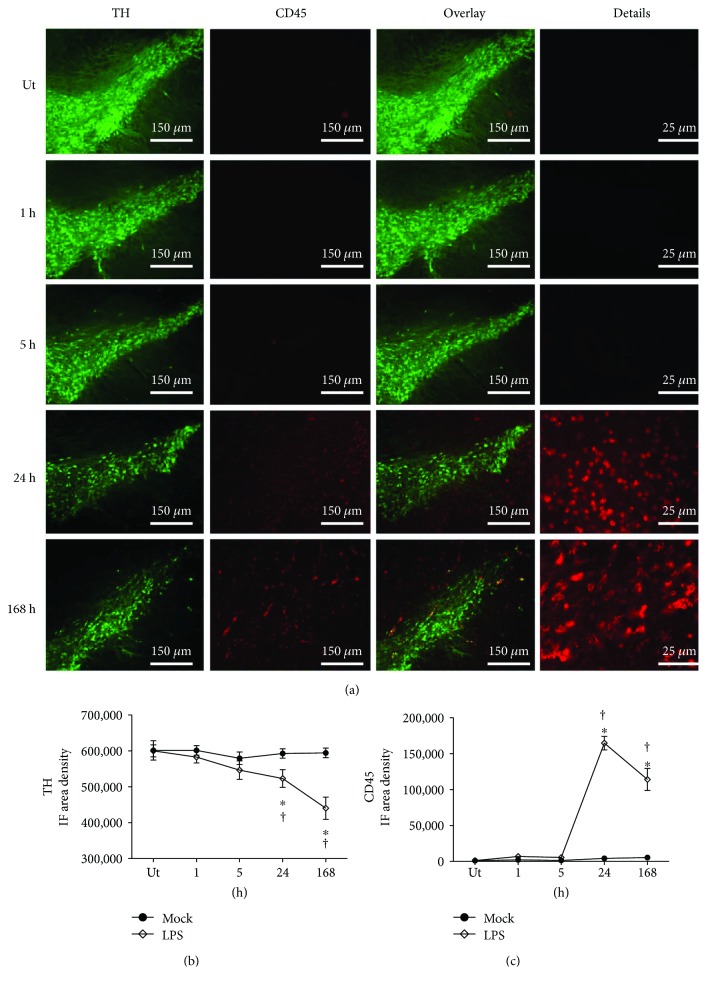
Time course of leucocyte infiltration. (a) Representative micrographs of the double immunofluorescence of CD45 and TH in the substantia nigra of untreated (Ut) control rats and rats at different times after LPS injection that were taken at 3.4 mm from the interaural midpoint on the dorsal-ventral axis of the rat brain atlas by Paxinos and Watson [50]. The numbers at the left side of micrographs indicate the time of evaluation. Immunofluorescence (IF) area density for TH (b) and CD45 (c) was determined using ImageJ software v.1.46r (National Institutes of Health, Bethesda, MD). The TH and CD45 values for the mock rats correspond to the quantification in Supplementary Figure 3. All values represent the mean ± SD (*n* = 3 rats for each time and for each experimental condition). ^∗^ *P* < 0.001 when compared with the untreated control group of the respective immunostaining. ^†^ *P* < 0.001 when compared with the respective mock group. Repeated-measures two-way ANOVA and Bonferroni post hoc test.

To further support leukocyte infiltration, immunostaining of CD45, a leucocyte common antigen [53], was performed in the substantia nigra (*n* = 3 rats in each time and experimental condition). The results show the absence of CD45^+^ cells in the untreated control and in the mock rats (Figure 11(c) and Supplementary Figure 3). In contrast, the presence of CD45^+^ cells is abundant at 24 h and 48 h after LPS injection (Figures 11(a) and 11(c)), as compared with those of their respective mock and untreated controls (Figure 11(c) and Supplementary Figure 3). These results show that infiltration of immunological cells predominates in the late phase of acute neuroinflammation.

## 4. Discussion

A leading line of research establishes that the microglial activation during neurodegeneration in the substantia nigra pars compacta is atypical [54]; that is, microglial activation leads to proinflammatory cytokine transcription but not translation [55]. This implies that microglial activation in certain conditions does not lead to an inflammatory response as believed. Experiments in a 6-OHDA Parkinson's disease model support the asseveration and truly extend the knowledge that proinflammatory cytokines such as TNF-*α* instead of being detrimental are beneficial for neurotoxin-induced neurodegeneration [56]. The studies on neuroinflammation in neurotoxin-induced Parkinson's disease models have been addressed in the critical period of neurodegeneration (7–21 days after neurotoxin injection) where the apoptotic process predominates [5, 19]. During this period, it is possible that a modulatory mechanism might be exerted on activated microglia to restrain cytokine translation, but not transcription, thus preventing their participation in neuroinflammation. In contrast, our results show that microglial activation by the archetypal inflammatory stimulus LPS can lead to transcription and translation of proinflammatory cytokines in a similar timeframe as that observed in macrophages activated during the innate immune response (local and systemic) [57–59]. Consecutively, astrocyte activation takes place and continues increasing until the end of the study (168 h after LPS injection). At this time, increased NO and lipid peroxidation levels, apparent phagocytosis of TH^+^ cells, and a significant decrease in TH immunoreactivity occur in the substantia nigra, thus suggesting the onset of neurodegeneration of dopaminergic neurons. The increase in NO already 2 h after the LPS stimulus suggests that NO production was independent of LPS-elicited proinflammatory cytokines. Based on these results, we propose that the alleged controversy on the involvement of activated microglia in neuroinflammation can be explained by the difference in the inflammatory stimulus used and the period where neuroinflammation variables are determined.

The increase in body temperature and sickness behavior induced by LPS show the systemic impact of pyrogenic cytokine production (TNF-*α*, IL-1*β*, and IL-6) in the substantia nigra. Our results agree with the results of previous studies showing that LPS-induced TLR4 signaling stimulates the synthesis of pyrogenic cytokines at the site of infection including the brain [42, 60]. The time course of fever and sickness behavior induced by the intranigral injection of LPS correlated with that of pyrogenic cytokines TNF-*α*, IL-1*β*, and IL-6 [42, 60]. It is interesting to note that the end of fever and sickness behavior coincides with the normalization of IL-6 levels, which is an important mediator of fever induction and a requisite in sustaining fever [60]. In this regard, the loss of IL-6 signaling is sufficient to abrogate fever in LPS- or IL-1-induced inflammatory models, even though TNF and IL-1 are increased in these settings [61–64]. Also, the time of defervescence of fever evoked by the intranigral injection of LPS coincided with the significant expression of IL-10, an antipyretic cytokine [65], in the substantia nigra. The mechanism of the anti-inflammatory effect of IL-10 is likely to be mediated through the inhibition of IL-1*β* which is locally produced [66]. This suggestion is supported by the finding that the increase in IL-10 expression accompanies the falling of IL-1*β* expression in the substantia nigra 24 h after LPS intranigral injection.

Microglial cells are resident macrophages of the CNS [67] and also bear TLR4, which can be activated by LPS to initiate an immune response entailing a wide range of immunomodulatory molecules such as proinflammatory cytokine and reactive oxygen species [68]. Since astrocytes are unresponsive to LPS [69], their activation depends on microglial NOX2-generated H_2_O_2_ that subsequently stimulates activation of transcription factors STAT1 and STAT3 [70]. This evidence indicates that microglial activation precedes astrocyte activation as supported by our results. We found that the peak of OX42 immunoreactivity was reached 24 h after LPS injection and was followed by a maximum increase in GFAP immunoreactivity 168 h after the LPS injection. At this latter time, a significant increase in NO concentration and lipid peroxidation was also present coinciding with the increased GFAP immunoreactivity. These results suggest that the generation of free radicals, mainly the radical H_2_O_2_, might participate in astrocyte activation. This phenomenon can be seen through the increased GFAP immunoreactivity, thickening of branches, and apparent mobility from the pars reticulata to the pars compacta of the substantia nigra shown by the detailed morphological analysis and the panoramic view of GFAP immunofluorescence.

The detailed morphological analysis also shows the prompt activation of microglia. The appearance of OX42 immunoreactivity and morphological changes is observed immediately after LPS injection. Five hours later, a combination of branched microglia and amoeboid microglia can be seen coinciding with the peak of proinflammatory cytokine production. These results suggest that at this time, the greatest transition state of active cells occurs. A predominant amount of amoeboid microglia can be seen 24 h after LPS injection, and it is possible that they also correspond to infiltrating macrophages attracted to the inflamed area by microglial chemokines as suggested by our CD45 immunostaining results at 24 h after LPS injection and the results of previous reports [8, 71]. This suggestion is further supported by the H&E staining that shows the presence of “rod” cells (characterized by the elongated and irregular nuclei and enlarged cytoplasm). Also, the H&E staining also supports leukocyte infiltration in the substantia nigra that can be attracted by microglial chemokines. At 168 h after LPS injection, the large and irregular OX42-immunoreactive cells resemble a phagocytic state of microglia. This suggestion is supported by the confocal orthogonal views that show microglial prolongations surrounding deteriorated dopaminergic neurons as if microglia were engulfing them for degradation. Also, previous studies have shown that this phenotype corresponds to phagocytic microglia when eliminating cellular debris [72].

Our results provide three pieces of evidence that sustain the degeneration of dopaminergic neurons in the substantia nigra at 168 h after LPS injection: (1) the decrease in TH immunoreactivity shown by TH immunofluorescence and immunohistochemistry assays that were performed together with glial markers (OX42 and GFAP) and H&E, respectively, (2) the irregular body contour and prolongation discontinuity of TH^+^ cells displayed by confocal microscope analysis, and (3) the phagocytosis of TH^+^ cells by OX42^+^ cells. The induction of neurodegeneration cannot be explained by a direct effect of LPS because dopaminergic neurons lack TLR4. Since nigral dopaminergic neurons are particularly vulnerable to nitrosative-oxidative stress [34], we propose that LPS-induced neuroinflammation is the cause of neurodegeneration.

## 5. Conclusions

Our results show that LPS evokes a typical acute inflammatory response in the substantia nigra of the rat (Figure 8). In this model of acute neuroinflammation, the microglial activation is the first event induced by LPS that is followed by astrocyte activation and leukocyte infiltration (Figure 8). Contrary to the “atypical” response observed in neurotoxin models of dopaminergic neurodegeneration, LPS leads to transcription and translation of proinflammatory cytokines at the initial phase of acute neuroinflammation, from 3 to 8 h (Figure 8). During this period, the increase in proinflammatory cytokine levels is associated with fever and sickness behavior (Figure 8). The acute increase in nitrosative-oxidative stress at the end of the period studied can favor neurodegeneration of dopaminergic neurons because of their susceptibility to neuroinflammation (Figure 8). While neuroinflammation in Parkinson's disease is chronic, our results in acute neuroinflammation can be useful to understand the progression of this disease.

## Supplementary Materials

Supplementary 1 Supplementary Figure 1: double immunofluorescence analysis against OX42 and TH in the substantia nigra of untreated (Ut) control rats and mock rats. The representative micrographs correspond to 3.8 mm from the interaural midpoint on the dorsal-ventral axis of the rat brain atlas by Paxinos and Watson [50]. The graph of immunofluorescence (IF) area density for TH and OX42 determined with ImageJ software v.1.46r (National Institutes of Health, Bethesda, MD) is shown in Figures 2(d) and 2(e), respectively. *n* = 3 independent rats in each time of each experimental condition.

Supplementary 2 Supplementary Figure 2: double immunofluorescence analysis against GFAP and TH in the substantia nigra of untreated (Ut) control rats and mock rats. The representative micrographs correspond to 3.8 mm from the interaural midpoint on the dorsal-ventral axis of the rat brain atlas by Paxinos and Watson [50]. The graph of immunofluorescence (IF) area density for TH and GFAP determined with ImageJ software v.1.46r (National Institutes of Health, Bethesda, MD) is shown in Figures 4(d) and 4(e), respectively. *n* = 3 independent rats in each time of each experimental condition.

Supplementary 3 Supplementary Figure 3: double immunofluorescence analysis against CD45 and TH in the substantia nigra of untreated (Ut) control rats and mock rats. The representative micrographs correspond to 3.4 mm from the interaural midpoint on the dorsal-ventral axis of the rat brain atlas by Paxinos and Watson [50]. The graph of immunofluorescence (IF) area density for TH and CD45 determined with ImageJ software v.1.46r (National Institutes of Health, Bethesda, MD) is shown in Figures 11(b) and 11(e), respectively. *n* = 3 independent rats in each time of each experimental condition.
